# Initial interaction of citrate-coated iron oxide nanoparticles with the glycocalyx of THP-1 monocytes assessed by real-time magnetic particle spectroscopy and electron microscopy

**DOI:** 10.1038/s41598-020-60162-8

**Published:** 2020-02-27

**Authors:** Wolfram C. Poller, Norbert Löwa, Moritz Schleicher, Agnieszka Münster-Wandowski, Matthias Taupitz, Verena Stangl, Antje Ludwig, Frank Wiekhorst

**Affiliations:** 1 Charité – Universitätsmedizin Berlin, corporate member of Freie Universität Berlin, Humboldt-Universität zu Berlin, and Berlin Institute of Health, Medizinische Klinik mit Schwerpunkt Kardiologie und Angiologie, Campus Mitte, Charitéplatz 1, 10117 Berlin, Germany; 2 Physikalisch-Technische Bundesanstalt, Abbestr. 2-12, 10587 Berlin, Germany; 3 Charité – Universitätsmedizin Berlin, corporate member of Freie Universität Berlin, Humboldt-Universität zu Berlin, and Berlin Institute of Health; Institut für Integrative Neuroanatomie, Charitéplatz 1, 10117 Berlin, Germany; 4 Charité – Universitätsmedizin Berlin, corporate member of Freie Universität Berlin, Humboldt-Universität zu Berlin, and Berlin Institute of Health; Institut für Radiologie, Campus Mitte, Charitéplatz 1, 10117 Berlin, Germany; 5 DZHK (German Centre for Cardiovascular Research), partner site Berlin, Berlin, Germany

**Keywords:** Biophysics, Biotechnology, Nanoparticles

## Abstract

Interaction with biological material can alter physicochemical parameters of magnetic nanoparticles and might thereby change their magnetic behavior with potentially important implications for various nanoparticle applications. Little is known about changes of the magnetic behavior that occur during the initial phase of cell binding and uptake. We investigate the magnetic behavior of very small superparamagnetic iron-oxide nanoparticles (VSOP) during initial contact with THP-1 monocytes. We combine real-time magnetic particle spectroscopy (MPS), a fast and sensitive method for specific detection of magnetic nanoparticles in biological specimen with high-pressure-freezing/freeze-substitution transmission electron microscopy (HPF/FS-TEM), enabling us to generate snapshots of the interaction of VSOP with the cellular glycocalyx. MPS reveals significant changes of the dynamic magnetic behavior within seconds after VSOP injection into monocyte suspensions that correlate with the formation of nanoparticle clusters in the glycocalyx. The combination of real-time MPS and HPF/FS-TEM provides an ideal platform to analyze magnetic behaviors of nanoparticles upon interaction with cells and tissues.

## Introduction

Magnetic iron oxide nanoparticles (MNP) serve in a broad spectrum of biomedical applications ranging from hyperthermia treatment of cancer over targeted drug delivery to contrast enhancement in magnetic resonance imaging (MRI) because of their special magnetic behavior in external magnetic fields^1^. This behavior is determined by the magnetic properties of MNP, which mostly depend on their composition, structure and size of the core. Additionally, physicochemical parameters such as surface coating and ζ potential as well as the mobility and aggregation state may further affect the magnetic behavior of MNP. As the interaction with biological material can alter the physicochemical parameters of the MNP their magnetic behavior may change as well^2–5^. While intermediate and long-term changes of MNP magnetism during the biodegradation process of cell-internalized MNP are relatively well investigated^6–9^, little is known about the change of the magnetic properties of MNP during the initial phase of cell contact and uptake.

Monitoring of the magnetic behavior of MNP during initial cell contact requires sensitive techniques with a high temporal resolution and the possibility to analyze living cells. Magnetic particle spectroscopy (MPS) was proven to be a fast, convenient and highly sensitive method for the specific detection of MNP in biological specimen^10,11^. By means of MPS the dynamic magnetic behavior of MNP under influence of an oscillating external magnetic field can be measured. The Fourier spectrum (An) of the measured temporal response is MNP specific and allows for sensitive quantification of MNP iron content, without being hampered by non-particular iron^11^. The amplitude and shape of the MPS spectrum is determined by many factors of influence. Apart from the main influential variables such as size distribution and magnetic anisotropy of the particles^12^, binding state^13^, viscosity^14^, and interparticle interactions^15^, also modulate the spectral signature of MNP in MPS. The slope of the MPS spectrum can be parameterized by the concentration independent ratio of the fifth and third harmonic (*A*_5_/*A*_3_ ratio)^5^. Changes of the dynamic magnetic behavior (expressed by a change in *A*_5_/*A*_3_ ratio) caused by aggregation, viscosity changes or biodegradation can be assessed in biological systems whereby the extent of signal change strongly depends on the initial MNP size^16^. Importantly, MPS enables analyses in living cells.

Using MPS we have previously observed changes of the dynamic magnetic behavior of citrate-coated very small superparamagnetic iron oxide nanoparticles (VSOP), which have entered the clinical development up to phase II trials as a contrast agent for MRI^17^, during incubation with THP-1 monocytes^2^. The human monocytic cell line THP-1 is widely used to study monocyte/macrophage biology in cell culture systems. Monocytes and macrophages are cells of the innate immune system that have been shown to incorporate VSOP in *in vivo* and *in vitro* experiments^2,18,19^. In this previous study, we analyzed washed cell-pellets with MPS and observed a significant increase in the A_5_/A_3_ ratio of VSOP three minutes after the start of cell incubation, followed by a steady decrease towards the baseline level over the next 24 h^2^.

Before an MNP can enter a cell, it has to pass the glycocalyx (GCX), a membrane anchored polymer layer consisting mainly of proteoglycans and hyaluronic acid. For a long time, this structure was neglected, but recently it has been recognized how decisive the GCX is for cellular bioprocesses (e.g. cell migration, proliferation, adhesion) and for the uptake of nanoparticles^20–22^.

In the current study, we therefore hypothesize that the interaction of VSOP with the GCX of THP-1 cells during their very initial contact causes physicochemical alterations of the nanoparticles that are responsible for rapid changes of their dynamic magnetic behavior, i.e. the increase of the *A*_5_/*A*_3_ ratio, after cell contact.

To test our hypothesis, we conducted real-time *in situ* MPS measurements during the first seconds and minutes of incubation of THP-1 cells with VSOP, to monitor changes of the *A*_5_/*A*_3_ ratio during the early VSOP-cell interaction without influencing the interaction by additional sample preparation steps. To visualize the association of VSOP with the GCX of THP-1 cells we used the technique of high-pressure-freezing/freeze-substitution transmission electron microscopy (HPF/FS-TEM), which was shown to largely preserve the hydrated configuration of the endothelial GCX^23,24^.

## Methods

### Very small superparamagnetic iron oxide particles (VSOP)

All chemicals and solvents were obtained from Sigma-Aldrich, Germany, unless otherwise stated. VSOP used in this study were synthesized as previously described in detail^2^. The mean hydrodynamic diameter of the nanoparticles (as measured by laser light scattering) was 8.7–11.0 nm with a polydispersity index of 0.085. The crystallite size measured by TEM (largest diameter of 500 crystals evaluated) was 6.8 ± 2 nm. The final ferrous iron ion content of 0.9% (molar ratio total iron) indicates nearly complete oxidation to maghemite. Selected area electron diffraction revealed a pure magnetite/maghemite pattern as previously published (PMID: 29017510).

### Cell culture and VSOP incubation

THP-1 cells (ATCC, Germany) were cultured in suspension in RPMI medium (1640; Invitrogen, Germany) supplemented with fetal calf serum (10% FCS, Biochrom, Germany), 100 U/mL penicillin (Invitrogen), 100 µg/mL streptomycin (Invitrogen*)*, as described previously^2^.

In a first set of experiments, 10^6^ THP-1 cells suspended in 100 µL of pre-warmed PBS were transferred to a glass tube and placed into the MPS spectrometer at 37 °C. After starting the MPS measurements, 100 µL of a VSOP solution in PBS were added through a syringe and mixed briefly by repeated pumping, while the glass tube remained in the spectrometer and was continuously measured by MPS (Bruker, Germany). The experiments were conducted in quintuplicate using a VSOP iron concentration of 0.5 mmol/L and 2.4 mmol/L, respectively. As control 100 µL VSOP with 0.6 mmol/L iron were added to 100 µL PBS without cells.

In a second set of experiments THP-1 cells were incubated with VSOP outside the spectrometer (*ex situ*), as described previously^2,18^. THP-1 cells (10 mL with 5·10^6^ cells/mL) were incubated with VSOP (0.5 mmol/L and 2.4 mmol/L). After incubation for 300 s, 600 s, and 900 s aliquots of 1 ml each were collected and centrifuged for 1 min at 500 g, and washed 2 times with PBS. Details of the analysis conditions are described below.

### Magnetic particle spectroscopy

Magnetic particle spectroscopy (MPS) measurements on VSOP samples were performed using a commercial magnetic particle spectrometer (MPS-3, Bruker, Germany) as previously described in detail^11^. Primarily, this device is dedicated to assess the performance of MNP as tracers for a novel imaging modality called magnetic particle imaging (MPI). MPS specifically detects the non-linear magnetic response of MNP exposed to an oscillating magnetic field. Therefore, biological tissue and paramagnetic blood iron do not contribute to the MPS signal.

MPS measurements in standard operation mode were performed as described elsewhere^6^. To investigate the MNP interaction with cells *in situ* real-time MPS measurements were performed. Therefore, 10^6^ cells diluted in 100 μL PBS were initially assembled in a glass tube (Bruker NMS PC 7.5). After placing the glass tube into the MPS pick-up coil repetitive measurements were started without VSOP to check for magnetic impurities. Over a time course of 10 min the MPS spectra were recorded every 4 s to improve the signal-to-noise-ratio. After the first measurements, 100 µL of VSOP with an iron concentration of *c*(Fe) = 0.5 mmol/L and 2.4 mmol/L were added stabilized in PBS and mixed thoroughly (5 times). To transfer the sample from the pipette to the bottom part of the glass tube a flexible tubing (Rotilabo-FEP, Carl Roth GmbH, Germany) was used to extend the tip. As a control the same procedure was applied to measure the time dependent behavior of 100 µL VSOP (*c*(Fe) = 0.6 mmol/L) after injection in medium (100 µL PBS) only without cells.

### High-pressure-freezing/freeze-substitution transmission electron microscopy

THP-1 cells were incubated with 1 mmol/l VSOP in RPMI medium supplemented with 10% fetal calf serum (FCS) for the indicated times (3 min, 30 min, 180 min) followed by centrifugation of 1 ml containing 10^6^ THP-1 cells (3000 rpm for 2 min at room temperature). The cell pellet was re-suspended in 20 µl RPMI medium, transferred into 3 mm copper gold-plated specimen carrier (200 µm indentation, Leica, Germany), coated with 1-hexadecene (Sigma-Aldrich, Germany) and immediately frozen in an EM ICE High Pressure Freezer (Leica, Germany) after reaching 2100 bar under liquid nitrogen. The detailed process of high-pressure freezing has been described previously^23^.

### Freeze-substitution and embedding

Frozen samples were collected in liquid nitrogen and transferred into a freeze-substitution chamber (Leica EM AFS; Leica, Germany), where they were freeze substituted for 12–14 hours at −90 °C in acetone containing 1% osmium tetroxide (OsO_4_; Electron Microscopy Sciences, Germany) to enhance contrast and simultaneously stabilize the glycocalyx. Subsequently, the temperature was gradually increased (5 °C/1 h) to 20 °C. Afterwards, the cells were centrifuged (1000 rpm, 3 min), washed with acetone (Roche, Germany) and centrifuged again. After rinsing several times with acetone, the dehydrated samples were infiltrated first with HPMA (2-hydroxypropyl methacrylamide; Sigma-Aldrich, Germany), centrifuged at 3000 rpm for 5 min and finally infiltrated overnight with HPMA-Epon mix (1:1). Suspension cells were further centrifuged at 5000 rpm for 5 min and infiltrated with pure freshly prepared Epon for several hours. Finally, the samples were embedded in Epon and polymerized overnight at 60 °C.

### Transmission electron microscopy

Ultrathin sections (60 nm) of embedded cells were cut using an ultramicrotome (Reichert Ultracut S, Leica, Germany) with a diamond knife (Diatome, Switzerland) and mounted on 300-mesh copper grids (Plano, Germany). Sections were examined, without further staining, using a Zeiss transmission electron microscope 912 (TEM-912, Carl Zeiss, Germany) operating at 80 kV and equipped with a digital camera (Proscan 2 K Slow-Scan CCD-Camera, Carl Zeiss, Germany). Digital image acquisition was performed using the iTEM software (Olympus GmbH, Germany)^2,18^.

### Data analyses

All MPS experiments were performed in quintuplicate, unless otherwise stated. Were appropriate, data are presented as mean ± standard deviations with a coverage factor of *k* = 1 (i.e. a confidence level of about 68%). The limit of detection was determined according to guidance of the International Union of Pure and Applied Chemistry (IUPAC) as mean + 3x standard deviation of empty sample holder measurements with a coverage factor of k = 1 as well.

## Results

### Analysis of pre-conditions for a real-time *in situ* MPS monitoring

**Figure 1:**
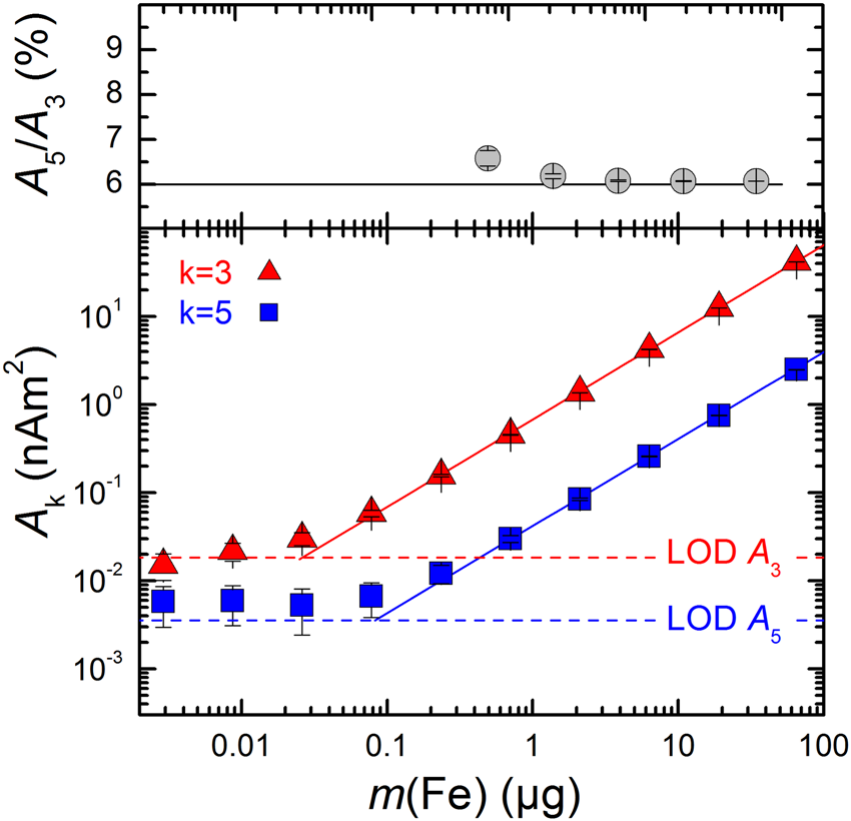
MPS measurements at $\hat{B}$ = 25 mT of VSOP in liquid suspension. MPS signals were acquired over a dilution range of the VSOP samples from 65 µg(Fe) down to 3 ng(Fe) iron. The uncertainty bars indicate the intrinsic noise of the MPS device determined by empty sample holder measurements (n = 100**)**. The upper graph shows the ratio of the third A3 to the fifth A5 harmonic amplitude representing the concentration independent slope of the MPS spectrum (grey circles) showing a mean A5/A3 ratio of 6.05% (solid line). The lower graph displays that the signal amplitudes *A*_3_ (red triangles) and *A*_5_ (blue squares) are decreasing linearly with sample iron content. The limit of detection (LOD) of the respective harmonic amplitude results from 20 repeated measurements of an empty sample holder (dashed lines).

We first analyzed in which concentration range VSOP can be detected by MPS. Therefore, serial dilutions of VSOP in liquid suspension (free VSOP) were measured by MPS and the limit of detection (LOD) of the third *A*_3_ and fifth *A*_5_ harmonic amplitude of the MPS signal was assessed. The amplitudes *A*_3_ and *A*_5_ of the MPS signal showed a highly linear dependency on the corresponding iron mass above their LOD of less than 200 ng(Fe) (Fig. 1). Furthermore, the shape of the MPS spectrum, represented by the ratio of the fifth *A*_5_ to the third *A*_3_ harmonic amplitude, remains constant within the examined concentration range at 6%. This further indicates that the dynamic magnetic behavior is not affected by changing concentrations of VSOP, which is a pre-condition for a real-time *in situ* MPS monitoring of VSOP during incubation with THP-1 cells. To detect the binding of VSOP to THP-1 cells as early as possible by real-time *in situ* MPS the addition of too much free VSOP should be avoided. Thus, the upper limit of *m*(Fe) was estimated to be 20 µg(Fe), which is the maximum VSOP uptake capacity of 10^6^ THP-1 cells^2^.

### Time resolved MPS measurements detect significant changes in the dynamic magnetic behavior of VSOP immediately after cell contact

**Figure 2:**
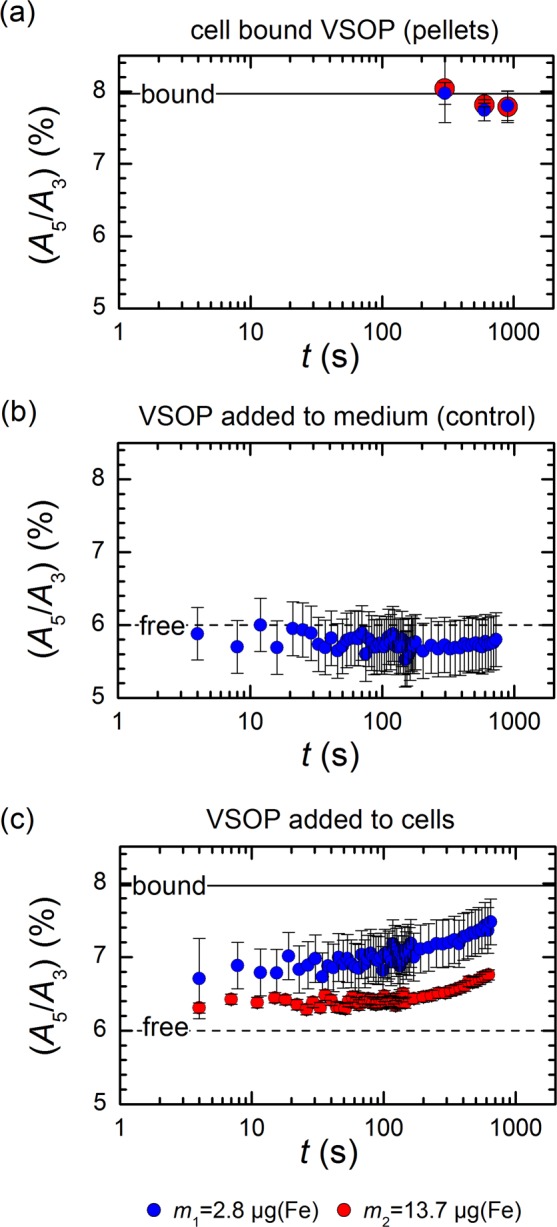
Cell binding and uptake of VSOP measured by MPS at $\hat{B}$ = 25 mT. **(a**) The mean and standard deviation of the MPS signal of cell pellets taken after 300 s, 600 s, and 900 s after start of VSOP incubation (n = 3). The *A*_5_/*A*_3_ ratio significantly increases for cell bound VSOP regardless of the iron amount used for incubation (blue and red circles). (**b**) Time dependent MPS signal of VSOP directly injected into medium only (PBS). The *A*_5_/*A*_3_ ratio (blue circles) does not change with time compared to the initial state (short dashed line). The uncertainty bars indicate the standard deviation of five replicated experiments (n = 5). (**c**) Time dependent MPS signal of VSOP directly injected into PBS with 10^6^ suspended THP-1 cells. A total iron amount of 2.8 µg (blue circles) and 13.7 µg (red circles) was used. A continuous increase of the *A*_5_/*A*_3_ ratio from the initial state to the bound VSOP state was observed depending on the applied iron amount. The uncertainty bars indicate the standard deviation of five replicated experiments (n = 5).

Figure 2a shows the results of *ex situ* MPS measurement of THP-1 cells after incubation with VSOP for 300 s, 600 s, and 900 s. The *A*_5_/*A*_3_ ratio of cell bound VSOP has increased to about 8% already after 300 s, irrespective of the VSOP concentration used for incubation. As the *A*_5_/*A*_3_ ratio resembles the magnetic properties of VSOP, this observation indicates rapid physicochemical changes during cell contact, which was reported previously for VSOP of a different formulation^2^.

In order to investigate the very first contact of VSOP with THP-1 cells, we took advantage of the capability of MPS to perform *in situ* real-time measurements at a minimum temporal resolution of 20 µs. Figure 2b displays the temporal evolution of the measured *A*_5_/*A*_3_ ratio after injection of VSOP into cell medium (PBS) only. The mean *A*_5_/*A*_3_ ratio of VSOP in PBS was 5.8% ± 0.2%, which is close to the *A*_5_/*A*_3_ ratio of free VSOP determined previously. This value remains nearly constant (coefficient of variation 2%) during the entire 12.5 minutes of real-time MPS scans. This control experiment proofs that VSOP were still freely mobile in the cell medium and the dynamic magnetic behavior of VSOP was not altered by the MPS measurement procedure itself (field amplitude, injection). Subsequently, a tube containing the THP-1 monocytes was placed into the MPS device and VSOP were directly added to this tube during ongoing MPS measurements. Figure 2c shows the resulting time course of the *A*_5_/*A*_3_ ratio, which was characterized by a continuous increase of the *A*_5_/*A*_3_ ratio for both amounts of added VSOP (*m*_1_ and *m*_2_). For the lower amount *m*_1_ of VSOP (filled blue circles) the *A*_5_/*A*_3_ ratio had already increased to 6.7% ± 0.5% within the first 4 s after addition to THP-1 cells. The mean *A*_5_/*A*_3_ ratio continuously increased throughout the entire incubation period and finally reached a value of 7.4% ± 0.3% after 12.5 minutes, which corresponds to an increase of about 31% from baseline. The injection of a higher amount *m*_2_ of VSOP resulted in a slightly lower change of the *A*_5_/*A*_3_ ratio (filled red circles), indicating a higher proportion of VSOP that remained unbound and freely mobile in the sample. With the higher amount *m*_2_, the *A*_5_/*A*_3_ ratio finally reached a value of 6.83% ± 0.07% (increase of 17% from baseline).

The quantified nanoparticular iron amount remained unchanged during the experiments as quantified by MPS and therefore a potential dissolution of the MNP can be excluded.

### Changes of the magnetic behavior of VSOP immediately after cell contact correlate with VSOP compartmentalization and clustering in the glycocalyx

**Figure 3:**
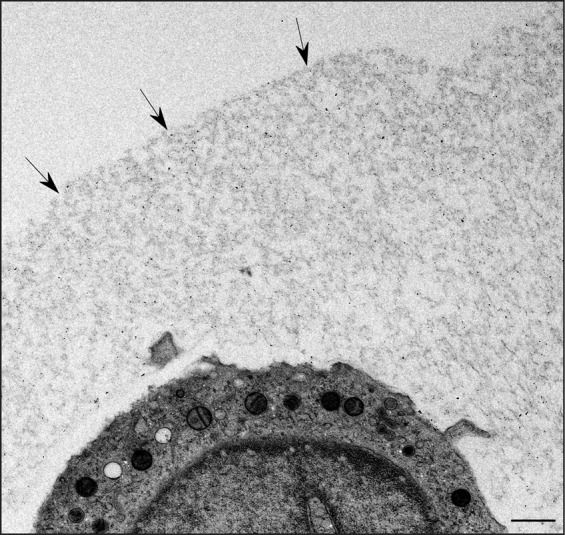
HPF/FS-TEM image showing the glycocalyx of a THP-1 monocyte after incubation with VSOP for 3 minutes. Black arrows indicate the outer border of the glycocalyx. Scale bar depicts 1 µm.

Imaging the interaction of the fragile glycocalyx with nanoparticles by TEM is challenging. Conventional sample preparation for TEM results in a nearly complete breakdown of the glycocalyx structure. By using HPF/FS-TEM we were able to visualize the glycocalyx of THP-1 cells as a several µm thick mesh-like structure (Fig. 3).

**Figure 4:**
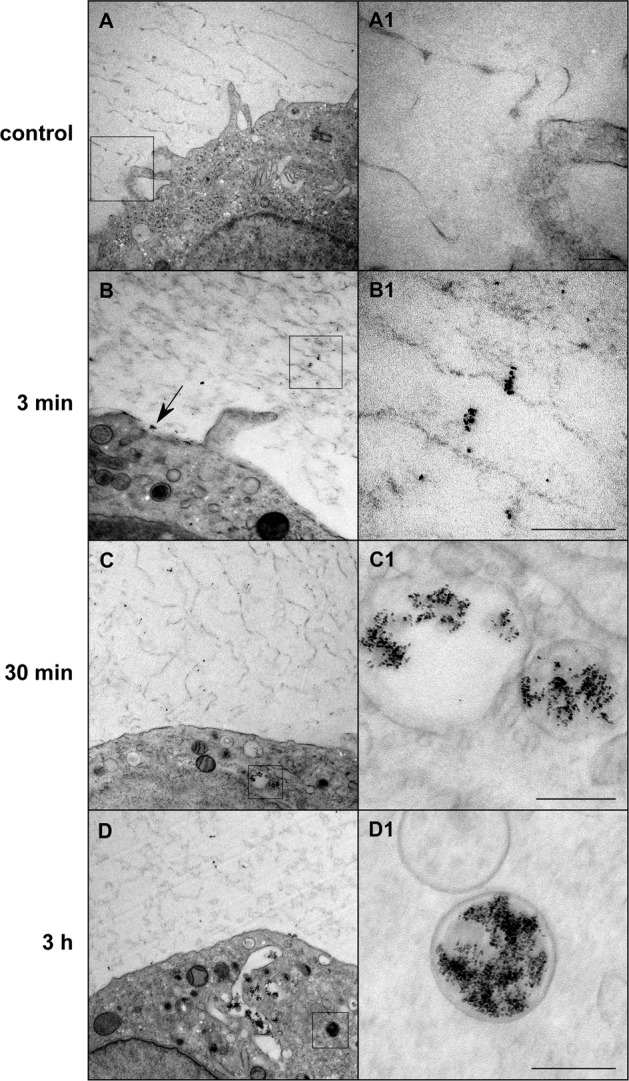
HPF/FS-TEM images showing the binding and uptake process of VSOP into THP-1 monocytes at the indicated incubation times. The control images A and A1 show a monocyte that was not incubated with VSOP. A1–D1 represent high-magnification images of the delineated area in **A**–**D**. VSOP are visible as black round structures with an individual nanoparticle diameter of 4–8 nm. The black arrow in B points to a small VSOP aggregate bound to the cell surface membrane. Scale bars depict 200 nm.

Figure 4 shows HPF/FS-TEM images of VSOP-labeled monocytes after the indicated incubation times at a high magnification to visualize the nanoparticles. At 3 minutes after the start of incubation, the majority of cell-bound VSOP were observed in small clusters distributed within the glycocalyx and as clusters directly on the cell membrane surface. Membrane invaginations associated with particle clusters indicated first endocytosis processes at this early time point of incubation. At later stages (30 minutes and 3 hours after incubation), the vast majority of cell-bound particles were found in cytoplasmic endosomes containing larger clusters of several hundred VSOP.

## Discussion

In this study we observed a significant change of the dynamic magnetic behavior of VSOP during the initial contact of VSOP with THP-1 monocytes that correlated with compartmentalization and clustering of the nanoparticles in the glycocalyx.

This was achieved by taking advantage of the capability of MPS to perform *in situ* magnetic analyses of magnetic nanoparticles in a suspension with living cells and to monitor conformational changes in real time. Due to the different spectral signature of free (dispersed) and cell bound (clustered) VSOP, we were able to analyze the temporal change of VSOP states during incubation with THP-1 monocytes. Therefore, we used the *A*_5_/*A*_3_ ratio, which serves as a concentration independent parameter for the spectral signature of VSOP in MPS and allows to monitor the transition process from free to cell bound VSOP.

We observed a continuous increase of the MPS harmonic ratio starting from the value of dispersed (free) up to clustered (cell bound) particles when VSOP were injected into cell suspensions. Conversely, no MPS signal change was measured after injection of VSOP into cell medium (PBS) only.

Interestingly, we observed a change of the spectral signature of VSOP in MPS towards higher values of the *A*_5_/*A*_3_ ratio, which is contrary to the commonly observed decrease of the *A*_5_/*A*_3_ ratio that occurs during progressive aggregation^3,4,25^ or cellular uptake^26,27^ of other MNP. This can be explained by the fact that such experiments were mostly performed with MNP of larger size and magnetic moment sufficient to deploy a rich harmonic spectrum in MPS, which is associated with a superior performance of these MNP in MPI. Magnetic dipolar interactions occurring between those large MNP in an aggregate lead to a reduced frequency response compared to non-interacting MNP, i.e. a lower spectral response and *A*_5_/*A*_3_ ratio in MPS. Due to the weak magnetic moment of the small sized VSOP, magnetic dipolar interactions have a positive effect on the frequency response. This was shown by Berkov *et al*. who found from numerical simulations that the magnetic dipolar interaction can either decrease or increase the frequency response of interacting MNP depending on the anisotropy of the individual MNP^28,29^.

Compared to our previously published experiments on washed pellets of VSOP-loaded cells^2^, we now detected significantly lower absolute values of the *A*_5_/*A*_3_ ratio both at baseline and after incubation with cells. As indicated by their MPS spectral signatures, these differences in the absolute values between the VSOP batch used in the previous study^2^ and the currently used one might be caused by a different size distribution of the nanoparticles observed with DLS (PDI 0.39 and 0.107 for previous batch and new batch, respectively) with a higher content of larger entities in the previous batch. Importantly, however, the relative increase in the *A*_5_/*A*_3_ ratio between the unbound stage and the cell-bound stage is strikingly similar around 30% for both VSOP batches (18.1% ± 0.1% to 24% ± 1% and 5.8% ± 0.2% to 7.4% ± 0.3% for previous batch and new batch, respectively)^2^, indicating that the same ultrastructural and magnetic alterations occurred.

Furthermore, the injection of a higher amount of VSOP resulted in a slightly lower change of the *A*_5_/*A*_3_ ratio. This indicates a higher proportion of VSOP that remained unbound and freely mobile in the sample. The observations from the *ex situ* experiments are in line with this hypothesis. For *ex situ* measurements, the cells were washed two times before the pellet was analyzed by MPS. These washing steps should have largely removed unbound nanoparticles, which have a lower *A*_5_/*A*_3_ ratio. Therefore, this approach leads to detection of the *A*_5_/*A*_3_ ratio of cell-bound VSOP only. Obviously, higher amounts of added VSOP do not seem to directly translate into higher amounts of VSOP in the glycocalyx. A potential explanation might be that VSOP only bind to specific domains within the glycocalyx or that changes in the electric charge distribution in the glycocalyx prevent further VSOP from entering the glycocalyx, so that only a certain amount of VSOP can be bound at a time. Another possibility is that, after a certain amount has been bound, it might just take more time for additional VSOP to enter the glycocalyx.

To correlate the observed MPS signal changes with morphological changes, we used the technique of high-pressure freezing with subsequent freeze substitution to fix our TEM samples. Chemical formaldehyde or glutaraldehyde based fixation methods inevitably induce strong ultrastructural changes that primarily effect water-rich labile components like the proteoglycans and glycosaminoglycans of the ECM, which is therefore almost completely lost during conventional fixation. HPF/FS-TEM overcomes these issues by simultaneously fixing all macromolecules in their hydrated biological configuration within milliseconds^23^. The rapid cooling process combined with the high pressure leads to the immediate formation of vitrified ice without any crystals^23^. In the subsequent freeze-substitution process, water is substituted by acetone and samples are slowly brought back to room temperature before the embedding process continues. Beside the much better ultrastructural preservation, HPF/FS-TEM also improves antigenicity for subsequent post-embedding immuno-labeling^23^. The major drawbacks of the method are its labor and cost expenses and the need for specific high pressure freezing and freeze substitution devices.

Using HPF/FS-TEM we were able to generate snapshots of the rapid and dynamic interaction of VSOP with the glycocalyx of THP-1 cells during the early cell contact^24^. For technical reasons the earliest time point that could be visualized was the state after 3 minutes. HPF/FS-TEM analyses revealed that the increase of the A5/A3 ratio temporally correlates with local clustering of VSOP in the glycocalyx of THP-1 cells in early phases, followed by the appearance of larger particle clusters in endosomes in later phases of VSOP incubation. As the MPS signal changed within seconds after injection of VSOP into cell suspension we suppose that VSOP clusters are formed in the glycocalyx immediately after initial contact.

The fast occurrence of VSOP clusters at the membrane and the prompt initiation of endocytosis of the particles, together with the results of real-time MPS point to a fast diffusion of a part of the VSOP through the glycocalyx without steric or electrostatic hindrance. Recent studies showed the size dependence of nanoparticle penetration into the glycocalyx and their accumulation there^20^. Lieleg *et al*. suggested a window of surface charges that enables a charged particle to diffuse within the glycocalyx without electrostatic hindrance^30^. With a hydrodynamic diameter of ~10 nm and a zeta potential of −25 mV^31^ VSOP are obviously able to diffuse quickly into the glycocalyx. On the other hand, the clusters that were visible at certain sites of the glycocalyx could indicate that at least some of the VSOP interact with or bind to structures of the glycocalyx, such as proteoglycans and glycosaminoglycans (GAG). One possible interaction would be a transchelation reaction in which the citrate coat of the cationic iron oxide core of the VSOP is replaced by a GAG coat as hypothesized earlier^2,18,32^. GAG are strong chelators due to their high number of sulfation and carboxy groups and such a reaction was recently also proposed for citrate-coated gold nanoparticles, for which a high affinity to hyaluronic acid has been observed^21^. The glycocalyx of THP-1 cells has not yet been investigated in detail, however previous observations indicate that it mainly contains highly-chelating sulfated chondroitin sulfate GAG^33^.

To the best of our knowledge this study is the first description of a real-time MPS approach for live analyses of interactions between MNP and cells. The combination of real-time MPS and HPF/FS-TEM provides an ideal platform to analyze interactions of different MNP with the glycocalyx of various cell types.

## References

[1] Krishnan, K. M. *Fundamentals and Applications of Magnetic Materials*. (Oxford University Press, 2016).

[2] Poller WC (2016). Magnetic particle spectroscopy reveals dynamic changes in the magnetic behavior of very small superparamagnetic iron oxide nanoparticles during cellular uptake and enables determination of cell-labeling efficacy. J. Biomed. Nanotechnol..

[3] Löwa N, Seidel M, Radon P, Wiekhorst F (2017). Magnetic nanoparticles in different biological environments analyzed by magnetic particle spectroscopy. J Magn Magn Mater.

[4] Arami H, Ferguson RM, Khandhar AP, Krishnan KM (2013). Size-dependent ferrohydrodynamic relaxometry of magnetic particle imaging tracers in different environments. Med Phys.

[5] Rauwerdink AM, Weaver JB (2010). Viscous effects on nanoparticle magnetization harmonics. J Magn Magn Mater.

[6] Löwa, N., Wiekhorst, F., Metzkow, S., Ludwig, A. & Trahms, L. Magnetic Particle Spectroscopy for the Quantification of Magnetic Nanoparticles in Living Cells. *Biomed Tech (Berl)* **58** Suppl 1, 10.1515/bmt-2013-4138 (2013). 10.1515/bmt-2013-4138 24042788

[7] Mazuel F (2016). Massive Intracellular Biodegradation of Iron Oxide Nanoparticles Evidenced Magnetically at Single-Endosome and Tissue Levels. ACS Nano.

[8] Mazuel, F. *et al*. In *Tissue Engineering Part A* Vol. 22 (ed. Inc Mary Ann Liebert) S107–S107 (2016).

[9] Poller WC (2018). Very small superparamagnetic iron oxide nanoparticles: Long-term fate and metabolic processing in atherosclerotic mice. Nanomedicine.

[10] Gleich B, Weizenecker J (2005). Tomographic imaging using the nonlinear response of magnetic particles. Nature.

[11] Löwa, N. *et al*. In *IEEE Trans Magn* Vol. 49, no. 1 275,278 (2013).

[12] Ludwig F (2013). Characterization of magnetic nanoparticle systems with respect to their magnetic particle imaging performance. Biomed Tech (Berl).

[13] Rauwerdink, A. & Weaver, J. Measurement of molecular binding using the Brownian motion of magnetic nanoparticle probes. *Appl. Phys. Lett*. **96** (2010).

[14] Draack S (2019). Multiparametric Magnetic Particle Spectroscopy of CoFe2O4 Nanoparticles in Viscous Media. J. Phys. Chem. C.

[15] Wu K, Su D, Saha R, Liu J, Wang J (2019). Investigating the Effect of Magnetic Dipole-Dipole Interaction on Magnetic Particle Spectroscopy (MPS): Implications for Magnetic Nanoparticle-based Bioassays and Magnetic Particle Imaging (MPI). arXiv.org> physics> arXiv.

[16] Teeman E, Shasha C, Evans JE, Krishnan KM (2019). Intracellular dynamics of superparamagnetic iron oxide nanoparticles for magnetic particle imaging. Nanoscale.

[17] Wagner M (2011). Coronary MR angiography using citrate-coated very small superparamagnetic iron oxide particles as blood-pool contrast agent: initial experience in humans. J Magn Reson Imaging.

[18] Ludwig A (2013). Rapid binding of electrostatically stabilized iron oxide nanoparticles to THP-1 monocytic cells via interaction with glycosaminoglycans. Basic Res Cardiol.

[19] Poller WC (2016). Uptake of citrate-coated iron oxide nanoparticles into atherosclerotic lesions in mice occurs via accelerated transcytosis through plaque endothelial cells. Nano Research.

[20] Xu R (2014). Pericellular matrix plays an active role in retention and cellular uptake of large-sized nanoparticles. Anal Bioanal Chem.

[21] Zhang S, Moustafa Y, Huo Q (2014). Different interaction modes of biomolecules with citrate-capped gold nanoparticles. ACS Appl Mater Interfaces.

[22] Zhou R, Zhou H, Xiong B, He Y, Yeung ES (2012). Pericellular matrix enhances retention and cellular uptake of nanoparticles. J Am Chem Soc.

[23] Vanhecke D, Graber W, Studer D (2008). Close-to-native ultrastructural preservation by high pressure freezing. Methods Cell Biol.

[24] Ebong EE, Macaluso FP, Spray DC, Tarbell JM (2011). Imaging the endothelial glycocalyx *in vitro* by rapid freezing/freeze substitution transmission electron microscopy. Arterioscler Thromb Vasc Biol.

[25] Rauwerdink AM, Weaver JB (2010). Harmonic phase angle as a concentration-independent measure of nanoparticle dynamics. Med Phys.

[26] Arami H, Krishnan KM (2014). Intracellular performance of tailored nanoparticle tracers in magnetic particle imaging. J Appl Phys.

[27] Antonelli A (2013). Red blood cells as carriers in magnetic particle imaging. Biomed Tech (Berl).

[28] Berkov DV, Gorn NL (2001). Susceptibility of the disordered system of fine magnetic particles: a Langevin-dynamics study. J Phys Condens Matter.

[29] Bervkov DV, Gorn NL, Görnert P (2002). Magnetization Dynamics in Nanoparticle Systems: Numerical Simulation Using Langevin Dynamics. Phys Status Solidi.

[30] Lieleg O, Baumgartel RM, Bausch AR (2009). Selective filtering of particles by the extracellular matrix: an electrostatic bandpass. Biophys J..

[31] Scharlach C (2015). Synthesis of acid-stabilized iron oxide nanoparticles and comparison for targeting atherosclerotic plaques: Evaluation by MRI, quantitative MPS, and TEM alternative to ambiguous Prussian blue iron staining. Nanomedicine.

[32] Wagner S (2013). Contrast-enhanced MR imaging of atherosclerosis using citrate-coated superparamagnetic iron oxide nanoparticles: calcifying microvesicles as imaging target for plaque characterization. International journal of nanomedicine.

[33] Ambrosius M, Kleesiek K, Gotting C (2008). Quantitative determination of the glycosaminoglycan Delta-disaccharide composition of serum, platelets and granulocytes by reversed-phase high-performance liquid chromatography. J Chromatogr A.

